# Lifestyle differences between older migrants and non-migrants in 14 European countries using propensity score matching method

**DOI:** 10.1007/s00038-017-1010-5

**Published:** 2017-07-13

**Authors:** Jelena Arsenijevic, Wim Groot

**Affiliations:** 1 Department of Health Services Research, Faculty of Health, Medicine and Life Sciences, CAPHRI, Maastricht University Medical Center, Maastricht University, PO Box 616, 6200 Maastricht, The Netherlands; 2 Top Institute Evidence-Based Education Research (TIER), Maastricht University, Maastricht, The Netherlands; 3 Faculty of Law, Economics and Governance, Utrecht University, Utrecht, The Netherlands

**Keywords:** Migration, Country of destination, Country of origin, European countries, Propensity score matching, Cross-sectional study

## Abstract

**Objectives:**

We examine the differences in lifestyle between four groups of migrants—first generation of older migrants originating from one of the EU countries, Africa or the Middle East and second-generation older EU migrants—with non-migrants in their country of destination.

**Methods:**

We use wave 5 of the SHARE data. To control for differences in socio-demographic characteristics, cultural factors and duration of stay in country of destination between migrants and non-migrants, we use propensity matching score analysis.

**Results:**

Older migrants from Southern European countries are more likely to smoke than non-migrants in their country of origin. Older migrants originating from Africa and the Middle East are more likely to smoke than non-migrants in their country of destination. Some groups of second-generation older migrants are more likely to consume alcohol and to have lower levels of physical activity than non-migrants in their country of destination.

**Conclusions:**

Our results show that differences in lifestyle between migrants and non-migrants exist, but they are not solely related to their migrant status. Cultural and socio-demographic characteristics also play a role.

## Introduction

With the increase in migration, the integration of migrants has become an important topic of debate. Lifestyle (smoking, alcohol consumption or a low level of physical activity) is an important area of integration (Darmon and Khlat 2001; Rechel et al. 2013). Of course, a healthy lifestyle also contributes to the life expectancy and health status of migrants and may reduce the pressure on the health care system in the country of destination (Mladovsky 2009; Rechel et al. 2013) (Reus-Pons et al. 2017).

Assessing differences in lifestyle between the migrants and non-migrants based only on migrant status can yield misleading conclusions about the effect of being a migrant on lifestyle (Heinrich et al. 2010). The reason for this is the problem of (self) selection—migrants may differ from non-migrants in both the country of origin and the country of destination in many observable and unobservable characteristics that also influence their lifestyle. These observable characteristics include socio-demographic characteristics (gender, age, educational and economic status), acculturation factors (cultural habits in country of origin, assimilation in country of destination) and duration of stay in country of destination (Borjas et al. 2015; Lee and Chung 2013).

To correct for this self-selection bias, previous studies have used a multitude of strategies. Some studies have controlled for socio-demographic characteristics—comparing the lifestyle of migrants with non-migrants with the same gender or the same education level. Those studies have reported both positive and negative differences between migrants and non-migrants (Borjas et al. 2015; Delavari et al. 2013; Dogra et al. 2010; Hosper et al. 2007; Koca and Lapa 2014; Lee and Chung 2013; Melchior et al. 2015; Nierkens et al. 2011; Riosmena et al. 2013; Schmidt et al. 2008). Another group of studies has controlled for bias arising from acculturation factors. Most of these studies focus on the lifestyle of Hispanic migrants coming to the US and compare them with non-migrants in their country of destination or their country of origin (Riosmena et al. 2013; Rubalcava et al. 2008). Those studies show that the prevalence of an unhealthy lifestyle is lower among Hispanic migrants than among non-migrants in the country of origin (Riosmena et al. 2013). A similar situation is observed when Hispanic migrants are compared with non-migrants in their country of destination—US. However, the longer the duration of the stay in the US, the unhealthier the lifestyle becomes. Similar findings have been observed for differences in mental health status between Hispanic migrants and non-migrants—a longer stay in the country of destination is a predictor for mental health problems (Alegría et al. 2007; Cook et al. 2009). Some of those studies also show that an unhealthy lifestyle (smoking or alcohol consumption) can be a trigger for mental health problems (Gonçalves and Cook 2016). In contrast, the few available studies for Europe show that there are no differences in lifestyle between migrants and non-migrants in their country of origin (Alves et al. 2013, 2015).

The main limitation of the previous studies is that they controlled only for bias arising from some socio-demographic or cultural characteristics using statistical models such as ordinary least square regressions (OLS). However, this is a naïve way to address the self-selection bias problem (Abramitzky et al. 2013). Recent studies have gone one step further. They have compared migrants with similar non-migrants based on all relevant observable characteristics using propensity score matching analyses (PSM) (Abramitzky et al. 2013; Lee and Chung 2013). Some studies examined the causal effect of migrant status on health applying an instrumental variable approach (Cullinan and Gillespie 2015; Johnson and Taylor 2012). However, those studies focussed on working migrants from countries outside Europe and on their general health rather than on lifestyle (Johnson and Taylor 2012; Lee and Chung 2013).

There are also studies that have ignored the problem of self-selection. They mostly report that the prevalence of an unhealthy lifestyle is higher among migrants than among non-migrants (Reiss et al. 2014; Urban et al. 2015). For example, Urban et al. compare smoking behaviour between migrants and non-migrants without taking into account other observable characteristics that might influence smoking like—for example—education. Simple descriptive statistical tests are used to compare the two groups.

As the results from the previous studies are rather mixed, they have also come up with different explanations for their findings. Positive lifestyle differences are usually explained by a “positive or healthy migrant effect”—migrants are those who are healthier, better educated and have a healthier lifestyle than non-migrants in the country of origin and country of destination (Johnson and Taylor 2012; Kennedy et al. 2015).

Economists have argued that migrants tend to migrate to countries where the rate of return to skills is higher which gives rise to positive selection bias (Borjas et al. 2015). This positive selection can contribute to positive differences in lifestyle between migrants and non-migrants (Lee and Chung 2013). Negative differences in lifestyle between migrants and non-migrants are also explained by the “positive migrant effect” with negative acculturation in health (Abramitzky et al. 2013). Although migrants have a healthy lifestyle in their country of origin, when staying in the country of destination they tend to adopt an unhealthy lifestyle (Kennedy et al. 2015). In addition, some studies explain negative differences by negative self-selection—a negative lifestyle such as smoking can predict future migration (Silventoinen et al. 2008). Those studies show that controlling only for socio-demographic characteristics or only for country of destination can lead to imprecise results and consequently produce ambiguity in the interpretation.

To accurately assess differences in lifestyle between migrants and non-migrants, this study takes into account observable socio-demographic characteristics, cultural factors and duration of stay in the country of destination. We use data from the Survey on Health and Retirement in Europe (SHARE), 5th wave with information on older migrants in 14 European countries. We distinguish four different groups of older migrants: first-generation migrants from Europe, Africa or Middle East countries and second-generation migrants from Europe. We compare migrants coming from one of the 14 European countries with non-migrants in the country of origin and the country of destination. To account for self-selection bias we use propensity score matching (PSM) to compare each migrant to a non-migrant with similar characteristics. In this way, this study bridges a gap in our knowledge about the lifestyle of migrants who live in European countries. This may help to develop appropriate programs to improve the lifestyle of older migrants.

## Methods

We use SHARE data wave 5 collected in 2013 in Austria, Belgium, Czech Republic, Denmark, Estonia, France, Germany, Italy, Luxembourg, the Netherlands, Slovenia, Spain, Sweden and Switzerland (Malter 2014). Respondents in SHARE are aged 50 and older. We include partners if they are born before 1962 (aged 50 and older). We distinguish first- and second-generation migrants (Börsch-Supan et al. 2013; Malter 2014). First-generation migrants are people born in another country than the country of residence. Second-generation migrants are individuals of whom at least one of the parents was born in another country. A detailed description of the SHARE data is available on SHARE web page (SHARE 2016).

We distinguish four groups of migrants. The first group is first-generation European migrants coming from one of the 14 European countries. They are compared with non-migrants in their country of origin and their country of destination. The second and third groups are migrants from Africa and Middle East countries, respectively. These represent the largest migrant communities in Europe.

The fourth group is second-generation migrants. We distinguish between those for whom the mother was a migrant and those for whom the father was a migrant from one of the 14 EU countries. The lifestyle of second-generation migrants is compared with non-migrants in country of destination. To compare second-generation older migrants with non-migrants, we exclude first-generation migrants to avoid potential bias. We assume that differences in lifestyle among second-generation migrants and non-migrants in their country of destination are no longer observed when other factors are controlled for.

The outcome variables are smoking, alcohol consumption and physical activity. Smoking is determined by the answer on the question “Do you smoke at the present time?”. Alcohol consumption is coded as 1, if the person drinks more than 2 glasses of alcohol per day (World Health Organization 2014), otherwise it is 0. If the person reports moderate physical activity, at least once per week, the physical activity variable is 1, otherwise it is 0.

We use PSM to create comparator groups. PSM allows us to compare each group of older migrants with a counterfactual group of older non-migrants. This ensures that the control group of non-migrants is most similar to the migrant group based on the chosen covariates. In this way, PSM corrects for the problem of selection bias. We also matched the group of migrants with the most similar group of non-migrants in their country of origin and/or country of destination. In this way, PSM corrects for self-selection bias.

PSM consists of several steps: identification of treatment and control group, identification of relevant covariates, calculation of propensity scores, matching the individuals from control and treatment groups based on their propensity score (PS) and estimating the treatment effects on outcome comparing the matched groups.

We first identify migrants and pair them in subsamples with non-migrants in their country of origin and/or their country of destination. For example, for all migrants who originate from Italy we create one subsample and pair them with all non-migrants in Italy and one subsample that pairs them with all non-migrants in their countries of destination.

Based on the existing literature we choose the following covariates for matching: age, gender, education level, marital status, work status, income status, body mass index, mobility status and general health status (Bouoiyour and Miftah 2015; Caliendo and Kopeinig 2008; Gilmore 1999; Lee and Chung 2013; Möllers and Meyer 2014; Warnes et al. 2004). Socio-demographic characteristics are most often used as covariates in PSM analyses on to migrant behaviour (Caliendo and Kopeinig 2008), body mass index and health status are also characteristics that influence lifestyle (Darmon and Khlat 2001; Gilmore 1999; Mulder et al. 1998). Physical activity is also related to mobility, especially among older adults (Koster et al. 2007).

For each subsample, we calculate the propensity score (PS). PS are estimated as the predicted probability of treatment conditional on the observed covariates (Austin 2011). In our case, the propensity score is a predicted probability using a regression on migrant/non-migrant status. To estimate the PS we use logistic regression applying STATA command psmatch2.

Based on the calculated PS we use a 1:1 matching method with no replacement to match each individual in the group of migrants with a similar individual in the group of non-migrants. Within each subsample for each number of migrants we have the same number of non-migrants with similar PS. For this purpose we use STATA commands psmatch2 and teffects psmatch. For each subsample, smoking, alcohol consumption and participation in physical activities is compared between matched migrants and non-migrants. For this comparison we estimate the average treatment effect on the treated (ATT). In case when all the characteristics of all individuals are equally distributed between the migrants and non-migrants (SB ≈ 0), ATT can be calculated as an average difference in outcome (in our case lifestyle) between the migrants and non-migrants:

$${\text{ATT}} = \frac{1}{N}\mathop \sum \limits_{i = 1}^{N} (Y_{i1} - Y_{i0} ),$$

where *Y*_*i*1_ is the average outcome of migrants and *Y*_*i*0_ is the average outcome of non-migrants and *N* is the number of matched individuals. For each group of migrants and for each subsample we present ATT results.

To check the robustness of our results, we also selected the 50% best matched pairs for each analysis and calculate the ATT for each of them.

## Results

The number of older individuals who originated from one of the 14 European countries is *N* = 792, which is approximately a third of all older migrants in our sample (*N* = 2656). The percentage of migrants originating from Africa or Middle East is 7.6 and 3.3%, respectively. Italy, France and Germany are most often reported as country of origin among older European migrants in our sample (Table 1), while Belgium, Luxembourg and Germany are the most frequent countries of destination. Germany and the Netherlands are interesting cases as the number of respondents who migrated to those countries is similar to the number of respondents who left them. Luxembourg is a destination for the majority of older migrants in our sample, while Estonia is not a chosen destination for any migrant in our sample. Among migrants originating from Africa, the most often reported countries of destinations are Belgium (25.9%), Luxembourg (16.7%), Spain (23.0%) and the Netherlands (10.5%) (Table 1). Migrants from Middle East countries most often choose Germany as the country of destination.

**Table 1:** Percentages of first (people who emigrate) and second generation of older migrants (people who are born in the country where at least one of the parent emigrated) based on their country of origin and country of destination (The Survey of Health, Ageing and Retirement in Europe, collected in Austria, Belgium, Czech Republic, Denmark, Estonia, France, Germany, Italy, Luxembourg, The Netherlands, Slovenia, Spain, Sweden and Switzerland, 2013)

Country (sample size per country)	Share of first-generation migrants in the 14 European countries (% of *N*)		Share of second-generation migrants in the 14 European countries (% of *N*)		Division of the first-generation migrants from outside Europe across European countries^c^ (% of *N*)	
	Country of origin (country from which migrants came from) *N* = 792	Country of destination (country where migrants live now)^a^ *N* = 792	Mother migrant *N* = 527^b^	Father migrant *N* = 353	Migrants who originate from Africa *N* = 239	Migrants who originate from Middle East *N* = 114
Austria	0.5	3.9	1.4	0.1	0.4	0.9%
Belgium	12.9	9.5	1.7	1.1	25.9	9.6%
Czech Republic	4.3	5.9	0.4	0.2	0.0	0.0
Denmark	3.3	1.6	0.2	0.4	0.8	8.8%
Estonia	0.0	0.4	0.7	0.1	0.0	0.0
France	0.8	15.8	1.0	0.1	2.9	0.0
Germany	23.3	26.9	0.1	1.0	1.3	33.3
Italy	1.0	17.9	0.2	0.2	0.8	0.9
Luxembourg	36.7	.6	0.0	8.1	16.7	2.6
The Netherlands	3.8	6.3	0.3	0.1	10.5	7.0%
Slovenia	1.4	4.2	0.4	0.4	0.0	0.0
Spain	5.3	3.9	0.1	0.1	23.0	0.0
Sweden	4.3	2.0	0.4	0.5	2.1	12.3%
Switzerland	2.3	1.0	5.3	0.3	0.0	0.0

^a^% of older migrants per country

^b^% of second generation per country of destination

^c^% of older migrants outside Europe based on *N*

Table 2 shows the prevalence of unhealthy behaviour among different groups of migrants. Smoking is most prevalent among first-generation European migrants who have migrated to Denmark, France and Switzerland, while alcohol consumption is more prevalent among those who have migrated to Belgium, Denmark and Luxembourg. Irrespective of their country of destination, the prevalence of physical activity varies from 60.0 to 91.2%.

**Table 2:** The incidence of unhealthy lifestyle behaviour among different groups of migrants and non-migrants (unmatched sample) (The Survey of Health, Ageing and Retirement in Europe, collected in Austria, Belgium, Czech Republic, Denmark, Estonia, France, Germany, Italy, Luxembourg, The Netherlands, Slovenia, Spain, Sweden and Switzerland, 2013)

	Non-migrants		First-generation migrants						Second-generation migrants		
			European migrants (origin)	European migrants (destination)		Originating from Africa		Originating from Middle East	Mother migrant		Father migrant
Smoking at the present time (percentage of older adults who reported smoking)											
Austria	19.8		12.9	0.0		0.0		0.0	25.4		0.0
Belgium	18.4		22.7	43.6		24.2		45.6	11.8		50.0
Czech Republic	22.8		12.8	29.0		0.0		0.0	0.0		30.0
Denmark	19.7		23.1	56.3		0.0		30.0	11.1		55.6
Estonia	19.1		0.0	0.0		0.0		0.0	27.5		0.0
France	15.5		19.5	50.0		0.0		0.0	18.2		0.0
Germany	19.6		13.9	29.5		0.0		28.9	25.0		37.5
Italy	14.9		21.8	20.0		0.0		0.0	28.6		33.3
Luxembourg	15.4		24.2	32.4		17.5		0.0	0.0		37.5
The Netherlands	16.2		20.0	38.9		24.0		25.0	35.7%		0.0
Slovenia	14.5		24.2	20.0		0.0		0.0	7.7		0.0
Spain	12.8		16.7	12.7		7.3		0.0	0.0		0.0
Switzerland	23.5		12.5	47.1		0.0		–	29.3		28.6
Sweden	12.2		37.5	21.1		40.0		21.4	11.8		33.3
Consuming more than two glasses of alcohol per day (percentage of the older adults who reported drinking more than two glasses of alcohol per day)											
Austria	11.6		12.9	0.0		0.0		0.0	8.5		0.0
Belgium	23.1		14.7	20.8		16.1%		0.0	15.1		13.8
Czech Republic	89.7		19.1	11.8		0.0		0.0	0.0		0.0
Denmark	29.6		20.8	38.5		0.0		30.0	33.3		27.3
Estonia	16.9		0.0	0.0		0.0		0.0	25.0		0.0
France	16.1		15.4	0.0		14.3		0.0	15.9		100.0
Germany	16.0		18.2	15.8		0.4		0.0	100.0		16.3
Italy	4.4		12.7	0.0		0.0		0.0	100.0		100.0
Luxembourg	16.3		15.2	20.1		27.5		0.9	0.0		18.3
The Netherlands	21.0		24.0	6.7		20.0		25.0	14.3		0.0
Slovenia	5.1		15.2	0.0		0.0		0.0	7.7		0.0
Spain	7.7		23.3	7.1		12.7		0.0	16.7		50.0
Switzerland	27.9		12.5	16.7		0.0		0.0	28.7		37.5
Sweden	17.2		27.5	8.8		20.0		14.3	17.6		5.9
Participating in moderate physical activity (percentage of older adults who reported to be engaged in physical activity)											
Austria	87.8		96.8	0.0		0.0		0.9	93.2		0.0
Belgium	86.0		96.0	84.9		77.4%		90.8	86.0		91.2
Czech Republic	89.3		91.5	91.2		0.0		0.0	0.0		90.9
Denmark	92.6		92.3	88.5		0.0		8.8	100.0		90.1
Estonia	85.7		0.0	0.0		0.0		0.0	92.5		0.0
France	87.2		91.1	60.0		14.3		0.0	93.2		100.0
Germany	90.6		88.5	89.1		1.3		78.9	100.0		88.4
Italy	76.9		82.4	62.5		0.4		0.9	50.0		83.3
Luxembourg	88.2		84.8	92.0		90.0		2.7	0.0		91.3
The Netherlands	90.9		92.0	93.3		88.0		100.0	85.7		0.0
Slovenia	87.6		84.8	90.0		0.0		0.0	84.6		0.0
Spain	81.9		93.3	87.8		72.7		0.0	100.0		100.0
Switzerland	87.1		87.5	83.5		0.0		0.0	86.6		75.0
Sweden	94.0		81.3	91.2		80.0		71.4	88.2		94.1

Among migrants from African countries, the prevalence of smoking is high and varies from 17.0 to 40.0% depending on the country of destination and the sample size. The prevalence of smoking is also high when comparing migrants from Africa with non-migrants in their countries of destination. Physical activity is lower among this group of migrants compared to non-migrants in their country of destination. This is contrary to findings on alcohol consumption—alcohol consumption among this group of migrants is lower than among non-migrants. The same pattern is observed when migrants originating from Middle East are compared with non-migrants in their country of destination.

Tables 3, 4, 5 and 6 present the differences in lifestyle among matched groups of first-generation migrants from EU countries and non-migrants in the country of destination and in the country of origin. Migrants are more likely to smoke in comparison with non-migrants when the country of origin is France (ATT = 0.13), Italy (ATT = 0.20) and Spain (ATT = 0.27). Migrants from Belgium smoke less than non-migrants in their country of origin (ATT = −0.06). On the other side, migrants from European countries who choose Belgium as their country of destination are more likely to smoke than non-migrants. Migrants who originate from Spain (ATT = 0.17) and Italy (ATT = 0.11) are more likely to be engaged in alcohol drinking than non-migrants in their country of origin.

**Table 3:** Differences in lifestyle behaviour among migrants from European countries and non-migrants in the country of destination (country of destination is one of the countries represented in The Survey of Health, Ageing and Retirement in Europe, collected in Austria, Belgium, Czech Republic, Denmark, Estonia, France, Germany, Italy, Luxembourg, The Netherlands, Slovenia, Spain, Sweden and Switzerland in 2013 where migrants live after migration)

Country of destination	ATT (smoking at present time)^a^			ATT (moderate sport activity)^a^			ATT (alcohol drinking)^a^		
	Treated	Controls	Differences	Treated	Controls	Differences	Treated	Controls	Differences
Belgium	0.42	0.22	0.20*	0.85	0.89	−0.05	0.21	0.29	−0.08
Chez Republic	0.26	.26	0.00	0.90	0.90	0.00	0.12	0.15	−0.03
Denmark	0.56	0.18	0.38	0.88	0.99	−0.11*	0.38	0.23	0.15
Germany	0.29	0.26	0.03	0.90	0.91	−0.01	0.16	0.18	−0.02
Luxembourg	0.32	0.36	−0.04	0.92	0.92	−0.00	0.20	0.14	0.06*
The Netherlands	0.38	0.44	0.05	0.93	0.93	0.00	0.06	0.26	−0.20*
Spain	0.12	0.25	−0.13	0.87	0.75	0.12	0.07	0.11	−0.04
Sweden	0.16	0.16	0.00	0.90	0.93	−0.03	0.09	0.18	−0.09

* Difference between migrants and non-migrants significant for *p* ≤ 0.05

** Difference between migrants and non-migrants significant for *p* ≤ 0.10

^a^ ATT = average treatment effect on treated calculated as a difference in lifestyle behaviour between the migrants (treated) and non-migrants in their country of destination (control); difference = treated − controls

**Table 4:** Differences in lifestyle behaviour among migrants from European countries and non-migrants in the country of origin (country of origin is one of the countries represented in The Survey of Health, Ageing and Retirement in Europe, collected in Austria, Belgium, Czech Republic, Denmark, Estonia, France, Germany, Italy, Luxembourg, The Netherlands, Slovenia, Spain, Sweden and Switzerland in 2013 where migrants came from)

Country of origin	ATT (smoking at present time)^a^			ATT (moderate sport activity)^a^			ATT (alcohol drinking)^a^		
	Treated	Controls	Differences	Treated	Controls	Differences	Treated	Controls	Differences
Austria				0.96	0.90	0.06			
Belgium	0.18	0.24	−0.06*	0.96	0.87	0.09*	0.14	0.26	−0.12*
Chez Republic	0.26	0.11	0.15	0.95	0.93	0.02	0.20	0.16	0.04
France	0.31	0.18	0.13**	0.90	0.91	−0.01	0.15	0.18	−0.03
Germany	0.25	0.29	−0.04	0.90	0.92	−0.20	0.18	0.17	0.01
Italy	0.36	0.16	0.20*	0.83	0.77	.06	0.12	0.01	0.11*
Luxembourg	0.30	0.34	−0.04	0.83	0.93	−0.01	0.16	0.03	0.13
The Netherlands	0.32	0.35	−0.035	0.93	0.91	0.02	0.24	0.32	−0.08
Spain	0.33	0.06	0.27*	0.93	0.86	0.06	0.23	0.06	0.17*

* Difference between migrants and non-migrants significant for *p* ≤ 0.05

** Difference between migrants and non-migrants significant for *p* ≤ 0.10

^a^ ATT = average treatment effect on treated calculated as a difference in lifestyle behaviour between the migrants (treated) and non-migrants in their country of destination (control); difference = treated − controls

**Table 5:** Differences in lifestyle behaviour among migrants from African countries and non-migrants in the country of destination (country of destination is one of the countries represented in The Survey of Health, Ageing and Retirement in Europe, collected in Austria, Belgium, Czech Republic, Denmark, Estonia, France, Germany, Italy, Luxembourg, The Netherlands, Slovenia, Spain, Sweden and Switzerland in 2013 where migrants live after migration)

	ATT (smoking at present time)^a^			ATT (moderate sport activity)^a^			ATT (alcohol drinking)^a^		
Country of destination	Treated	Controls	Differences	Treated	Controls	Differences	Treated	Controls	Differences
Belgium	0.55	0.29	0.26*	0.16	0.49	−0.32*	0.77	0.91	−0.14*
Luxembourg	0.41	0.41	0.00	0.90	0.95	−0.05	0.27	0.15	0.12
France	0.00	0.33	−0.33	–	–	–	0.33	0.16	0.17
The Netherlands	0.60	0.60	0.00	0.95	0.86	0.09	0.22	0.22	0.00
Spain	0.07	0.15	−0.08**	0.72	0.70	0.02	0.12	0.09	0.03
Sweden	0.50	0.00	0.50*	–	–	–	0.20	0.40	−0.20

* Difference between migrants and non-migrants significant for *p* ≤ 0.05

** Difference between migrants and non-migrants significant for p ≤ 0.10

^a ^ATT =  average treatment effect on treated calculated as a difference in lifestyle behaviour between the migrants (treated) and non-migrants in their country of destination (control); difference = treated − controls

**Table 6:** Differences in lifestyle behaviour among migrants from Middle East countries (first generation of migrants older than 55 coming from one of Middle East countries) and non-migrants in the country of destination (country of destination is one of the countries represented in The Survey of Health, Ageing and Retirement in Europe, collected in Austria, Belgium, Czech Republic, Denmark, Estonia, France, Germany, Italy, Luxembourg, The Netherlands, Slovenia, Spain, Sweden and Switzerland in 2013 where migrants live after migration)

Country of destination	ATT (smoking at present time)^a^			ATT (moderate sport activity)^a^			ATT (alcohol drinking)^a^		
	Treated	Controls	Differences	Treated	Controls	Differences	Treated	Controls	Differences
Belgium	0.83	0.33	0.50*	1.00	0.80	0.20	0.00	0.30	−0.30*
Denmark	0.50	0.25	0.25	1.00	1.00	0.00	–	–	–
Germany	0.52	0.42	0.10	0.78	0.94	−0.16*	0.00	0.16	−0.16*
The Netherlands	0.66	0.65	0.01*	–	–	–	0.25	0.37	−0.12
Sweden	0.30	0.30	0.00	0.71	.85	−0.14	0.14	0.28	−0.14

* Difference between migrants and non-migrants significant for p ≤ 0.05

** Difference between migrants and non-migrants significant for *p* ≤ 0.10

^a^ATT = average treatment effect on treated calculated as a difference in lifestyle behaviour between the migrants (treated) and non-migrants in their country of destination (control); difference = treated − controls

Tables 3, 4, 5 and 6 also show the differences in lifestyle between migrants originating from Africa and Middle East countries and non-migrants in their country of destination. Older migrants originating from Africa are more likely to smoke than non-migrants, if their country of destination is Belgium (ATT = 0.26) or Sweden (ATT = 0.50). However, they are more likely to have lower alcohol consumption in comparison with non-migrants if their country of destination is Belgium (ATT = −0.14) or Sweden (ATT = −0.20). Differences in smoking among migrants originating from Middle East and non-migrants are observed if the country of destination is Belgium (ATT = 0.50) or Denmark (ATT = 0.25).

Table 7 presents the differences in lifestyle among matched groups of second-generation migrants from EU countries and non-migrants in the country of destination. Second-generation migrants are more likely to consume alcohol if their country of destination is Switzerland and the mother is a migrant from Germany (ATT = 0.10) or Austria (ATT = 0.18). Similar findings are observed if the country of destination is Czech Republic (father migrant from Germany) and Austria (father migrants from Czech Republic). Second-generation migrants are also more likely to engage in physical activity when the country of destination is Austria (father migrant from Germany, ATT = 0.06), Chez Republic (mother migrant from Austria, ATT = 0.12) and Luxembourg (mother migrant from Germany, ATT = 0.14), but opposite results are observed in Switzerland (mother migrant from France, ATT = −0.12 and mother migrant from Spain, ATT = −0.22). In Switzerland, second generation of migrants is less engaged in physical activity than non-migrants.

**Table 7:** Differences in lifestyle behaviour among second-generation migrants from European countries (migrants are born in the country of destination while one of the parent is born in the country different than country of destination) and non-migrants in the country of destination (country of destination is one of the countries represented in The Survey of Health, Ageing and Retirement in Europe, collected in Austria, Belgium, Czech Republic, Denmark, Estonia, France, Germany, Italy, Luxembourg, The Netherlands, Slovenia, Spain, Sweden and Switzerland in 2013 where migrants live after the migration)

	ATT (smoking at present time)^a^				ATT (moderate sport activity)^a^					ATT (alcohol drinking)^a^			
	Treated	Controls		Differences	Treated		Controls	Differences		Treated	Controls		Differences
*Country of destination Austria*													
Father migrant Germany	0.15	0.19		−0.04	0.093		0.087	0.06**		0.11	0.09		0.02
Father migrant Chez Republic	0.19	0.22		−0.03	0.85		0.77	0.08**		0.14	0.13		0.01*
*Country of destination* *Belgium*													
Father migrant Italy	0.19	0.24		−0.05	0.84		0.83	0.01		0.17	0.27		−0.10*
Father migrant Germany	0.23	0.29		−0.06	0.83		0.94	−0.11**		0.05	0.16		−0.11
Mother migrant Italy	0.20	0.28		−0.08**	0.83		0.78	0.05		0.18	0.16		0.02
*Country of destination Chez Republic*													
Mother migrant Austria	0.30	0.26		0.04	0.96		0.84	0.12*		0.02	0.02		0.00
Mother migrant Germany	0.06	0.17		−0.11	0.95		0.95	0.00		0.15	0.45		−0.30*
Father migrant Germany	0.28	0.38		−0.10	0.91		0.91	0.00		0.26	0.09		0.17*
*Country of destination France*													
Mother migrant Belgium	0.10	0.08		0.02	0.78		0.92	−0.13*		0.21	0.18	0.03	
*Country of destination* *Germany*													
Father migrant Chez Republic	0.32	0.32		0.00	0.92		0.91	0.01		0.03	0.12	−0.09*	
*Country of destination* *Luxembourg*													
Mother migrant France	0.42	0.21		0.21*									
Mother migrant Germany	–	–		–	0.98		0.84	0.14*		–	–	–	
*Country of destination* *The Netherlands*													
Mother migrant Belgium	0.18	0.36		−0.18**	–		–	–		–	–	–	
Father migrant Germany	0.23	0.13		0.11**	–		–	–		–	–	–	
*Country of destination* *Sweden*													
Father migrants Denmark	0.37	0.12	0.25*		–	–		–	–		–	–	
*Country of destination* *Switzerland*													
Father migrant Austria	0.43	0.18	0.25*		–	–		–	–		–	–	
Father migrants France	0.25	0.36	−0.11*		–	–		–	–		–	–	
Mother migrants France	–	–	**–**		0.83	0.95		−0.12*	–		–	–	
Mother migrant Spain	–	–	–		0.76	0.99		−0.22*	–		–	–	
Mother migrant Germany	–	–	–		–	–		–	0.31		0.21	0.10*	
Mother migrant Austria	–	–	–		–	–		–	0.36		0.18	0.18*	

* Difference between migrants and non-migrants significant for *p* ≤ 0.05

** Difference between migrants and non-migrants significant for *p* ≤ 0.10

^a^ ATT = average treatment effect on treated calculated as a difference in lifestyle behaviour between the migrants (treated) and non-migrants in their country of destination (control); difference = treated − controls

## Discussion

Our results show that differences in lifestyle between migrants and non-migrants exist, but that the relation between migrant status and lifestyle is not straight forward. Those differences are not only related to migrant status, but also to their country of origin, country of destination and type of lifestyle. Differences in smoking behaviour and alcohol consumption are observed among all three groups of first-generation migrants, but their direction differs and depends on the country of origin and country of destination. The first generation of European migrants smokes more than non-migrants in their country of origin. However, differences in smoking are only observed when migrants who originate from South European countries (France, Italy and Spain) are compared with non-migrants in their country of origin. The majority of the migrants from South European countries move to higher income countries, so-called “not egalitarian” countries where the rate of return to skills is higher. This includes countries like Denmark, Belgium and Luxembourg (see Table 1). This means that those migrants can more afford to smoke than non-migrants in their country of origin. Differences in smoking between migrants and non-migrants in their country of destination are not observed. Those findings are not only in accordance with the economic explanation of migration but also with a “healthy migrant effect”. According to the healthy migrant effect, migrants are those who are healthier, better educated and have a healthier lifestyle than non-migrants in the country of origin and country of destination.

Migrants originating from Africa and the Middle East smoke more than non-migrants in their country of destination. These differences are more likely a result of a lack of integration of those migrants. For example, smoking may be socially acceptable in the country of origin for those groups of migrants. Therefore, smoking is rather a way of preserving the values of the country of origin. Besides the fact that those groups of migrants might be more eager to preserve the values and lifestyles of their countries of origin, they may also face problems such as stress at work, stress caused by language barriers or stress provoked by the fact that they are separated from their families which may make them more likely to smoke (Alegría et al. 2007; Gonçalves and Cook 2016).

After matching, we find positive or no differences in alcohol consumption between migrants originating from Africa and the Middle East countries and non-migrants in the country of destination. The differences in alcohol consumption among first-generation European migrants can be both positive and negative.

Nevertheless, differences in alcohol consumption are more obvious among migrants from Africa and the Middle East than among first-generation European migrants (Teuscher et al. 2015). In case of migrants from Africa and the Middle East, religion and social norms from the country of origin play a role in their lifestyle (Hosper et al. 2007). However, European migrants share a similar cultural background with non-migrants in their country of destination and differences in smoking and alcohol consumption among them are lower. Differences in physical activity between three groups of migrants and non-migrants are not observed. One possible reason is the fact that the physical activity scale includes sport activities and also other activities such as walking or working in the garden. However, it is not clear whether migrants and non-migrants are equally engaged in all types of activities.

We have also compared second-generation European migrants and non-migrants in their country of destination. Contrary to our expectation, some groups of second-generation older migrants are more likely to consume alcohol or to have lower levels of physical activity than non-migrants in their country of destination. Furthermore, differences in lifestyle among second-generation migrants are more often observed than among first-generation migrants. This means that social protection measures and health promotion interventions should address acculturation factors over a longer period of time.

The heterogeneity in results for the four groups of migrants and their lifestyle after adjusting for selection bias gives a more accurate insight into the lifestyle of migrants in general. Our results show that both the assumption of a “healthy migrant effect” and the economic explanation of migration cannot be solely used to explain differences in lifestyle between migrants and non-migrants. In a nutshell, our results show that for an accurate understanding of unhealthy behaviour among migrants, factors such as country of origin, economic reasons for migration and health policy towards migrants in the country of destination should be taken into account. The majority of migrants tend to use curative health care in the country of destination, while health promotion activities are usually neglected (Mladovsky 2009). The reasons for this may lie in a lack of tailor-made activities for migrants in the country of destination. For example, only some European countries (the Netherlands, Sweden and Germany) organize health promotion activities for migrants. However, even where those activities exist, migrants sometimes lack the knowledge and awareness about them. Furthermore, implementation of health programs also has some drawbacks. Programs for smoking cessation in migrant’s native languages even if successful may have a negative impact on the integration process (learning the language). Improving the use of health promotion activities among migrants may positively affect migrants’ lifestyle (Abma and Heijsman 2013; Teuscher et al. 2015; Verhagen et al. 2013).

This study has some limitations. When we compare the lifestyle of older migrants with non-migrants in their country of destination, we do not distinguish the migrants’ country of origin. This was not possible since the subsamples would become too small. Comparing each group of migrants with non-migrants in their country of origin and their country of destination can give better insight into differences in lifestyle. In this study for consistency we have compared only European migrants from one of the 14 countries. This can also have influenced the results.
